# Incidence of tuberculosis and its predictors among under-five children with severe acute malnutrition in North Shoa, Amhara region, Ethiopia: a retrospective follow-up study

**DOI:** 10.3389/fped.2023.1134822

**Published:** 2023-05-19

**Authors:** Yared Asmare Aynalem, Lemma Getacher, Yonatan Eshete Ashene, Tadesse Yirga Akalu, Getachew Yideg Yitbarek, Fanos Yeshanew Ayele, Dawit Aklilu, Emmanuel Akwasi Marfo, Tamiru Alene, Wondimeneh Shibabaw Shiferaw

**Affiliations:** ^1^ College of Health Science, Debre Berhan University, Debre Berhan, Ethiopia; ^2^ College of Health Sciences, Faculty of Nursing, University of Alberta, Edmonton, AB, Canada; ^3^ Department of Nutrition, North Shoa Zone Health Office, Amhara Regional Health Bureau, Debre Berhan, Ethiopia; ^4^ College of Health Science, Debre Markos University, Debre Markos, Ethiopia; ^5^ Department of Biomedical Sciences (Medical Physiology), College of Health Sciences, Debre Tabor University, Debre Tabor, Ethiopia; ^6^ School of Public Health, College of Medicine and Health Sciences, Wollo University, Dessie, Ethiopia; ^7^ Injibara University, College of Medicine and Health Science, Department of Pediatrics and Child Health Nursing, Injibara, Ethiopia; ^8^ School of Public Health, Faculty of Medicine, University of Queensland, Brisbane, QLD, Australia

**Keywords:** children, severe malnutrition, tuberculosis, Ethiopia, North Shoa

## Abstract

**Introduction:**

Although tuberculosis (TB) is one of the significant public health challenges in severely malnourished children throughout the globe, it is a severe issue for countries such as Ethiopia, with significant resource limitations. Few studies have examined the incidence of tuberculosis and its predictors among children under five years of age with severe acute malnutrition in developing countries, and there is a paucity of data. This study aimed to estimate the incidence of tuberculosis and its predictors among under-five children with severe acute malnutrition (SAM) in North Shoa, Amhara region, Ethiopia.

**Methods:**

An institution-based retrospective follow-up study was conducted between January 20, 2017, and June 20, 2019. The sample size was calculated using STATA, which yields a total of 345 charts that were selected with systematic random sampling. Data entry was performed using Epi-data version 4.2 and analyzed with STATA 14. Kaplan-Meier survival curves were computed. Cox proportional hazard models were fitted to detect the determinants of tuberculosis. The hazard ratio with a 95% confidence interval was subsequently calculated. Variables with *p*-values < 0.05 were considered statistically significant.

**Results:**

The incidence rate of tuberculosis among children under five years of age with SAM was 4.6 per 100 person-day observations (95% CI: 3.29, 8.9). Predictors of TB were a history of contact with known TB cases [AHR: 1.4 (95% CI: 1.00, 2.8], HIV/AIDS [AHR: 3.71 (95% CI: 2.10, 8.71)], baseline pneumonia [AHR: 2.10 (1.76,12)], not supplying zinc at baseline [AHR: 3.1 (1.91, 4.70)], and failed appetite taste at the diagnosis of SAM [AHR: 2.4 (1.35, 3.82)].

**Conclusions:**

In this study, the incidence rate of TB was high. Not supplying zinc at baseline, failed appetite taste at the diagnosis of SAM, history of contact with known TB cases, and baseline pneumonia were significant predictors of TB. Prioritizing regular TB screenings, nutritional support, and zinc supplementation for under-five children with SAM should be implemented to reduce the risk of TB.

## Introduction

Tuberculosis (TB) is among the most common determinants of infection-related morbidity and mortality worldwide. It is among the ten critical causes of childhood death globally. About 10 million people are diagnosed with TB worldwide; of those, 1 million are children (1). The World Health Organization (WHO) report shows that nearly 1.3 million deaths were attributed to TB (2, 3). Different studies have also revealed that childhood TB and undernutrition are the leading global problems, particularly among under-five children in resource-limited nations (4–7). Due to immune suppression, children with severe acute malnutrition (SAM) are more at risk of contracting infectious diseases, including TB, significantly contributing to high mortality (8, 9). Either TB or SAM is an independent contributing factor for high levels of morbidity and mortality among children across sub-Saharan South Africa (10–13).

Several socioeconomic factors, such as poor housing (14), poverty (15, 9) and economic deprivation, are associated with an increased risk of childhood TB infection (16). In addition, the link between tuberculosis and malnutrition has long been identified (17–19). TB causes loss of appetite, cachexia and wasting and may be impacted by poor nutritional status (20, 21). It also leads to worse nutrition and suppresses immunity undernutrition, thereby increasing the probability that latent TB will develop into an active disease (22–24). In addition, 3-fold also increases the risk of acquiring TB among malnourished children, which could predispose the community to progress to TB in high TB-burden settings, including Ethiopia (24–26). There is a decline in TB incidence and prevalence in all WHO counties, but the decline was not fast enough to achieve the 2020 target of ending the TB Strategy (4).

Different findings worldwide have revealed that the rate of TB is higher in malnourished children than in well-nourished children. For example, a report from some Asians revealed that the proportion of TB among malnourished children ranges from 8.8%–22% in India (29, 30). Similarly, a finding in African courtiers showed that the proportion of TB among SAM children varied from 1.58% in Zambia, 4.2% in Kenya (27), and 20% in Freetown (28).

According to the 2019 Mini Ethiopian DHS report, 38% of under-five children are stunted, while 10% are wasted, contributing to TB's uneven effect (26). Different findings from Ethiopia reported that two out of three registered TB patients are malnourished (31, 32). Studies conducted in Ethiopia also showed that the prevalence of TB among SAM varied from 6.8% in Pawi General Hospital ((35), 9.5%, and 9.68% in Gondar and 20.8% in Bahir Dar city. The TB spectrum in a cohort of pediatric patients with the specific combination with SAM contributed to the late achievement of the strategy and has not yet been well studied (14, 15). Hence, all TB care providers must integrate nutritional assessment and care intervention packages for all TB patients (33, 34).

Moreover, interest in the potential benefits of nutritional supplementation has also reemerged due to the geographic overlap between TB and undernutrition epidemics in many parts of the developing world, including Ethiopia (16, 17). However, despite this solid epidemiological linkage between malnutrition and TB infection in most sub-Saharan African countries, the incidence of TB among severely malnourished children needs to be better defined, particularly in the study area. Therefore, this study aimed to assess the incidence of tuberculosis and its predictors among hospitalized, severely malnourished under-five children in North Shoa Public Hospitals.

## Methods

### Study area, period, and design

An institution-based retrospective cohort study was conducted among children under five years of age with SAM admitted to the stabilizing center of a public hospital between January 20, 2017, and June 20, 2019, in North Shoa, Northeast Ethiopia. The Zone has a total population of 2.6 million with 90 health centers, one referral hospital and eight district hospitals. Sixteen stabilizing centers (two district hospitals and fourteen health centers) offer inpatient service and an isolation room for suspected TB cases. Nearly 467 children with SAM are treated in the pediatric ward annually. Physicians, health officers and nurses treat SAM based on the national protocol (18).

### Patient and public involvement

Patients and the public were not involved in the design, conduct, reporting, or dissemination plans of this research.

### Population and eligibility criteria

All charts of under-five children who were admitted to the stabilizing (therapeutic) feeding unit in North Shoa Public Hospital were the source population of this study. Moreover, the study population was the under-five children admitted to the stabilizing (therapeutic) feeding unit in the selected health institution. In addition, all records of under-five children with SAM admitted to stabilizing centers (SCs) were included. However, children with incomplete records and admission and discharge dates were excluded.

### Study variables

The incidence of TB among children with SAM was the outcome variable. On the other hand, sociodemographic characteristics (age, sex, residence, history of TB contact and admission characteristics, common comorbidity and treatments given to children) were the independent variables.

### Operational definition

**Time to TB diagnosis**: The time from admission with SAM and the development of TB.

**Event:** The occurrence of TB.

**Censored:** under-five children with SAM who were not developing TB until the end of the study died and were lost to follow-up during the study period.

**SAM:** Presence of any of the following WHO criteria: very low weight for height (Below −3z scores of the median/WHO growth standards), visible severe wasting, or presence of nutritional edema.

**TB:** children with at least one sign or symptom (Persistent (> two weeks), non-remitting cough, weight loss/failure to thrive, persistent (> one week), unexplained fever (>38°C) objectively recorded at least once, persistent, unexplained lethargy or reduced playfulness. Suggestive of TB and microbiologically confirmed TB, defined as at least one positive culture with M. TB speciation from a specimen representative of the intrathoracic disease. In addition, a history of fever and cough of over two weeks duration, failure to gain weight, loss of appetite, the decline in weight and symptoms of extrapulmonary tuberculosis such as lymphadenopathy, seizure, abdominal pain and history of contact with an open case of tuberculosis were recorded. Additionally, confirmed physician diagnoses were also used by reviewing their chart.

### Sample size determination and sampling procedure

The sample size of the current study was decided as all udder-five children with SAM who were admitted to the SC unit of the hospital between January 20, 2017, and June 20, 2019, in North Shoa, Northeast Ethiopia, and fulfilled the inclusion criteria of the study were considered. During the study period, approximately 467 clients were admitted to the SC unit of the hospital. The sample size was calculated using STATA as follows: Assumptions: 5% significant level, 80% power, survival probability in exposed (35%) and non-exposed (25%), survival probability (0.5), the proportion of withdrawal (8%), and exposed-to-non-exposed ratio (1:1). Finally, 345 were selected with systematic random sampling. After extensive evaluation of study participants by data collectors from the hospital charts, approximately 345 samples were finally included. In addition, study participants were selected from the registration data based on their entry time to the SC clinic.

### Data collection procedure

A data collection tool was prepared from the national treatment protocol for the management of SAM (18), SAM registration booklet, health management information system (HMIS) register, SAM multi-chart, TB guideline (19) and by reviewing articles (10, 20–22). The checklist used consisted of sociodemographic data (age, sex, residence, history of TB contact), anthropometric measurements (height, Mid Upper Arm Circumference (MUAC), weight, edema), comorbidities, feeding phase and types of feeding (F75, F100), frequency of feeding and amount per feed, immunization status, admission and discharge date, referral address as well as medication given and outcomes of the treatment. Nutritional status was assessed by weight, height or length, weight for height, and left upper-mid arm circumference. The follow-up period was from January 2014 to June 20, 2019. The first day of admission to treatment was taken as the starting follow-up time, while the last day of an event or censored occurrence was taken as the end of the follow-up time. The survival time was calculated by subtracting the first admission date from the last date of death or censoring. Four data collectors (BSc nurses) and two supervisors were recruited based on their experience in the SAM management process. Data collectors received one day of training on the tool and were only deployed to collect data once the principal investigator was convinced of their competency. The study's primary investigator and supervisors critically followed the data collection process to minimize missing information and inconsistencies.

### Data processing, analysis, and presentation

**Figure 1:**
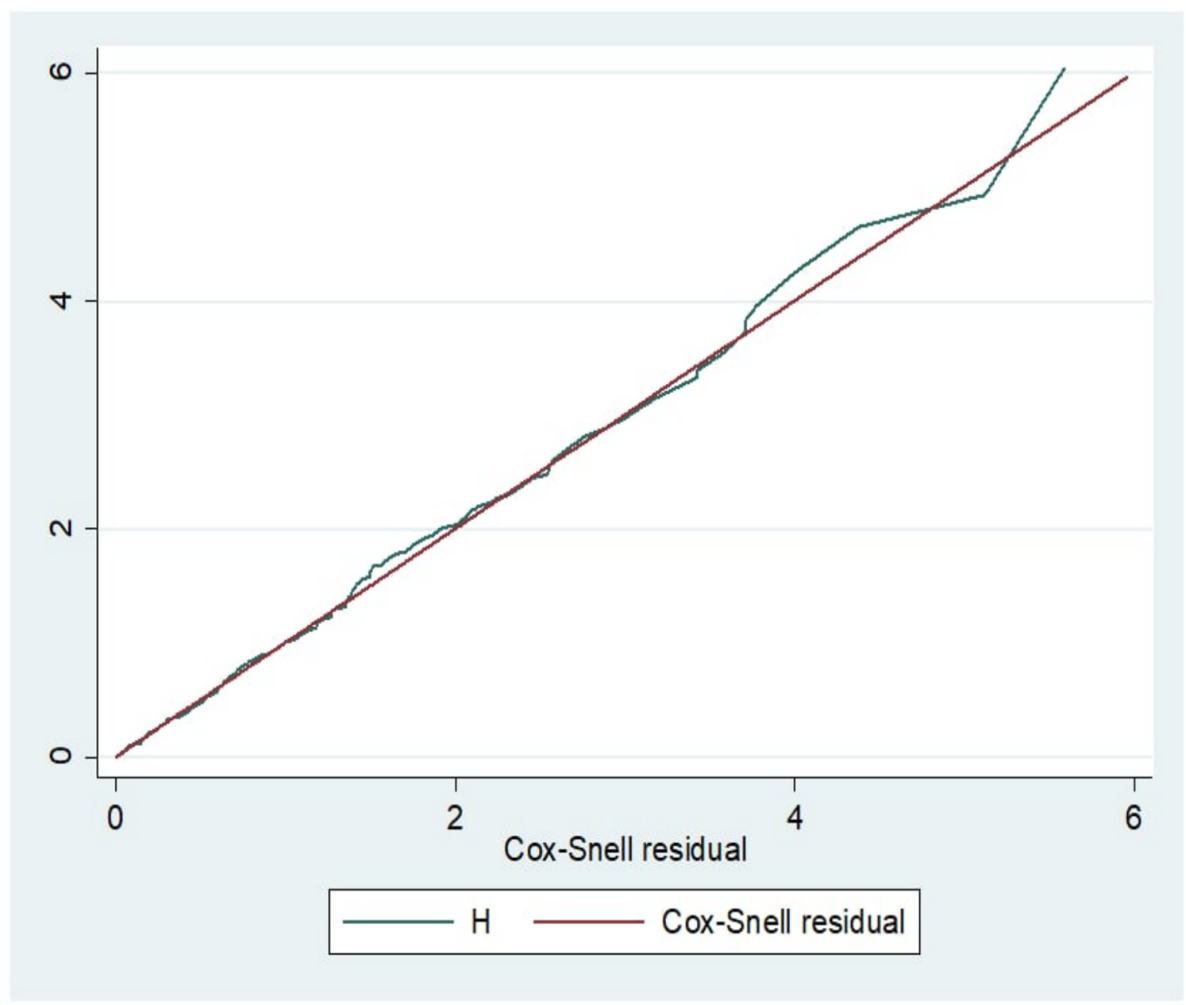
The Cox-snell residual Nelson-aalen cumulative hazard graph of TB among children with SAM in north shoa, amhara region, Ethiopia.

Data were cleaned, edited, and coded before analysis. Any errors identified at this time were corrected after reviewing the original data using the code numbers we had assigned during the data collection period. Data were entered using Epi-Data version 4.2.0 and analyzed using STATA 14 statistical software. Descriptive statistics were computed for categorical variables. First, the incidence density rate (IDR) was calculated for the entire study period. Subsequently, the number of TB cases within the follow-up period was divided by the total person-time at risk on follow-up and reported per 100-person months. Next, the data's normality was checked using the Shapiro-Wilk test. The outcome of each participant was dichotomized into censored or developing TB, and the incidence density rate was calculated for the entire study period. Kaplan-Meier curves with log-rank tests were used to estimate the median survival time and the cumulative probability of survival and compare survival times between two or more groups. Next, the Cox proportional hazard regression proportional hazard assumptions were checked using the Schoenfeld residual test. The test revealed that the assumption was met with a *p*-value of the global test = 0.45. The Cox-Snell residuals test also checked the model goodness-of-fit. The graph shows that the hazard function follows the 45-degree line very closely over time. This indicated that the final model was fitted (Figure 1). After checking the multicollinearity & interaction term, each independent predictor variable with a *p*-value < 0.25 in the bivariate analysis was included in multivariate Cox proportional hazard regression. Finally, an adjusted hazard ratio (AHR) with a 95% confidence interval and *p*-values < 0.05 was used to measure the strength of the association and identify statistical significance.

## Results

### Sociodemographic and admission characteristics

In the current study, 345 participants were included in the final analysis. Nearly one-third of the study subjects were female, and two hundred seventeen (62.9%) of the participants were below the age of 24 months. Regarding residential status, sixty-four percent of the participants came from rural areas. Concerning the feeding pattern, 185 (53.3%) children had a history of exclusive breastfeeding, and 120 (34.8%) had a bottle-feeding history. One hundred twenty-nine (37.4%) of the children had a history of contact with known TB cases (Table 1).

**Table 1:** Baseline sociodemographic and admission characteristics of children with tuberculosis in SAM admitted between January 20, 2014, and June 20, 2019, in north shoa, -and Ethiopia.

Baseline Variables	Category		Status	
		Frequency (%)	TB (%)	Censored (%)
Age	< 24 months	217 (62.9)	18 (8.3)	199 (91.7)
	≥24 months	128 (37.1)	6 (4.7)	122 (95.3)
Residency	Urban	123 (35.6)	9 (7.3)	114 (92.7)
	Rural	222 (64.4)	15 (6.8)	207 (93.2)
Sex	Male	211 (61.2)	13 (6.6)	198 (93.4)
	Female	134 (38.8)	11 (8.2)	123 (91.8)
Immunization status	Immunized	227 (65.8)	6 (2.6)	221 (97.4)
	Not immunized	118 (49.2)	18 (15.5)	100 (84.5)
Type of SAM	Edematous	241 (69.8)	16 (6.6)	225 (93.4)
	No edematous	104 (30.2)	8 (7.7)	96 (92.3)
Bottle feeding	No	225 (65.2)	20 (8.9)	205 (91.1)
	Yes	120 (34.8)	4 (3.3)	116 (96.7)
Exclusive breastfeeding	No	161 (46.7)	6 (3.7)	154 (96.3)
	Yes	185 (53.3)	18 (8.7)	167 (90.3)
Appetite test	Pass	215 (62.3)	3 (1.4)	212 (98.6)
	Failed	130 (37.7)	21 (16.1)	109 (83.9)
Level of consciousness	Conscious	259 (74.2)	14 (5.4)	245 (94.6)
	Unconscious	86 (25.8)	10 (11.6)	76 (88.4)
History of contact with TB patient	Yes	129 (37.4)	18 (13.9)	111 (86.1)
	No	216 (62.6)	6 (2,8)	210 (97.2)

SAM, sever acute malnutrition, TB, Tuberculosis.

### Common comorbidities

**Figure 2:**
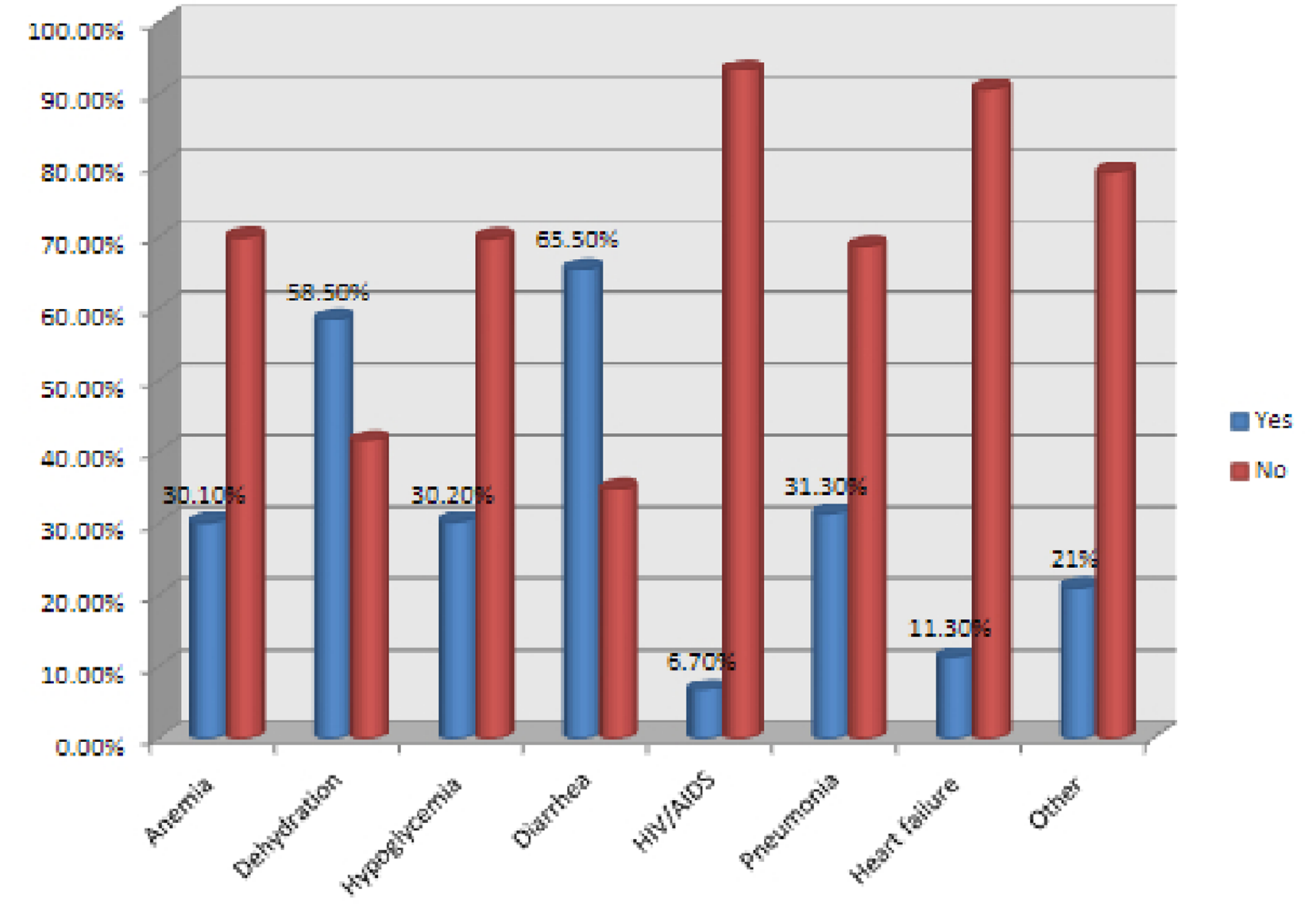
Common comorbidities of children with tuberculosis in SAM admitted from January 20, 2014, to June 20, 2019, north shoa, and Ethiopia. HIV/AIDS, Human Immunodeficiency Virus/Acquired Immunodeficiency Syndrome.

Baseline diarrhea, dehydration, pneumonia, hypoglycemia, and anemia were the common comorbidities, with proportions of 65.5%, 58.5%, 31.3%, 30.2% and 30.1%, respectively. The proportion of TB in under-five children with SAM was most common in pneumonia (18.5%), anemia (15.4%) and HIV/AIDS (15%) patients (Figure 2).

### Treatments are given to children

In the current study, 68.4% and 64.6% of subjects were given iron and F75, respectively. Seventy-four percent of the included children had a history of BCG vaccination. A high proportion of TB was reported among children who were transfused with blood (25%), not immunized with BCG (23.1%) and those who did not take PO antibiotics (15%) (Table 2).

**Table 2:** Baseline treatments given for under-five children with SAM admitted between January 20, 2014, to June 20, 2019, in north shoa, Ethiopia, and Ethiopia.

Baseline Variables	Category	Frequency (%)	Status	
			TB (%)	Censored (%)
F75	Yes	223 (64.6)	7 (3.2)	216 (97.7)
	No	122 (35.4)	17 (13.9)	105 (86.1)
F100	Yes	104 (30.1)	6 (5.9)	98 (94.1)
	No	241 (69.9)	16 (6.6)	225 (93.4)
Vitamin A	Yes	160 (46.3)	10 (6.3)	150 (93.7)
	No	185 (53.7)	14 (7.6)	171 (92.4)
Folic Acid	Yes	117 (33.9)	6 (5.1)	111 (94.9)
	No	228 (66.1)	18 (7.9)	210 (92.1)
Zinc	Yes	48 (13.9)	3 (6.3)	45 (93.7)
	No	297 (86.1)	21 (7.1)	276 (92.9)
Iron	Yes	237 (68.7)	16 (6.7)	221 (93.3)
	No	108 (31.3)	8 (7.4)	100 (92.6)
NG tube feeding	Yes	91 (26.4)	15 (16.5)	76 (83.5)
	No	254 (73.6)	9 (3.5)	245 (96.5)
Blood transfusion	Yes	28 (8.1)	7 (25)	21 (75)
	No	317 (91.9)	17 (5.4)	300 (94.6)
ReSoMal solution	Yes	90 (26.2)	16 (17.7)	74 (82.3)
	No	255 (73.8)	8 (3.1)	247 (96.9)
IV antibiotics	Yes	116 (33.6)	4 (3.5)	112 (96.5)
	No	229 (66.4)	20 (8.7)	209 (91.3)
Antibiotics	Yes	225 (65.2)	6 (2.7)	219 (97.3)
	No	120 (34.8)	18 (15)	102 (85)

IV, intravenous; NG, nasogastric.

### Incidence of TB in children with SAM

**Figure 3:**
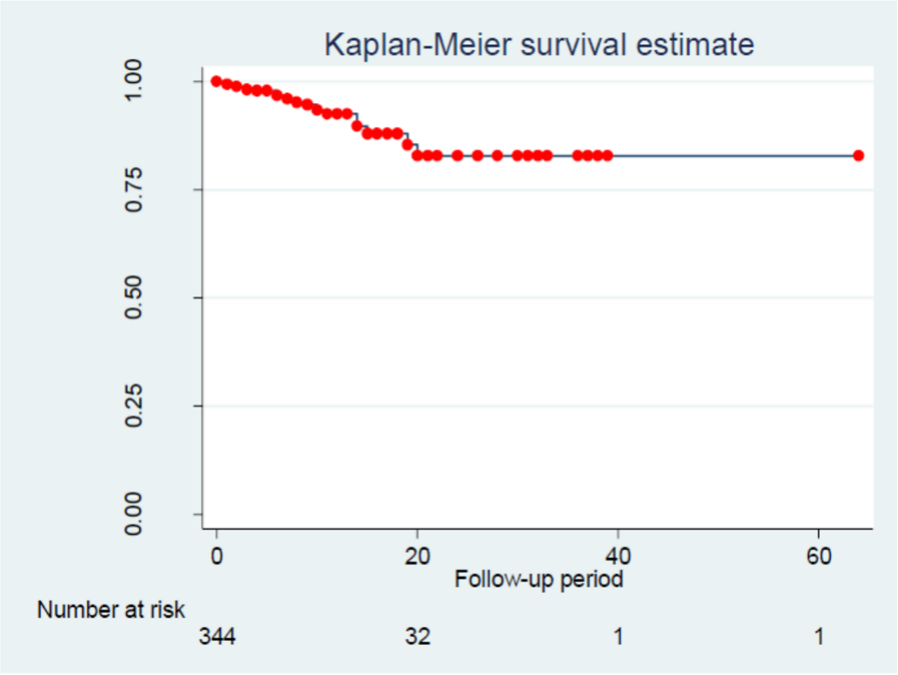
Overall kaplan-meier survival estimate TB among children with SAM in north shoa, amhara region, Ethiopia.

**Figure 4:**
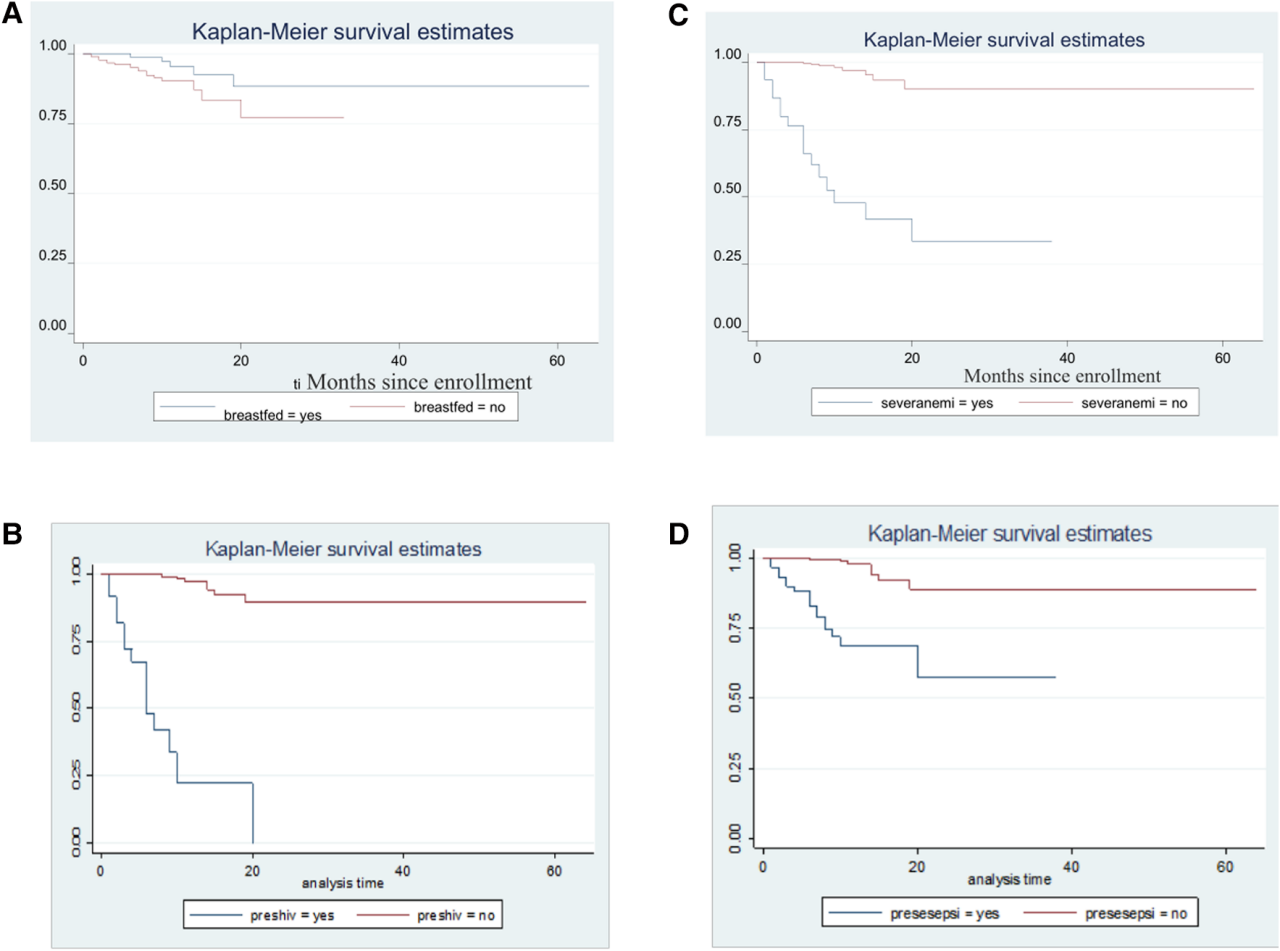
Kaplan-Meier survival estimates for time to TB occurrence among SAM children with respect to (**A**) breastfeeding status, (**B**) HIV/AIDS, (**C**) anemia and (**D**) sepsisA.

The overall cumulative incidence of TB was 7% (95% CI: 5.3–9.1). Likewise, the overall incidence density of TB among under-five children with SAM was found to be 4.6 per 100 child months (95% CI: 7.29, 8.9) with 3,723 person-months of observation. In this study, the estimated TB-free survival was 99.4%, 92.3% and 82.9% at 1, 13 and 24 months, respectively (Figure 3). The proportional hazard assumption was evaluated using Kaplan-Meier survival curves and the Sheffield residual global test and was found to be met (x^2^ = 4.11; *p*-value = 0.08) (Figure 4).

### Predictors of time to TB occurrence

After controlling for confounding factors with multivariable Cox regression analysis, no supplementation zinc at the base line of the study, failed appetite taste at the diagnosis of SAM, history of contact with known TB cases, HIV/AIDS and baseline pneumonia were significant predictors of TB in under-five children with SAM. Accordingly, children with a history of contact with known TB cases had a 1.4 times higher risk of contracting TB than their counterparts [AHR: 1.40 (95% CI: 1.11,2.81)]. Likewise, children who did not have zinc supplementation as part of the SAM treatment had a 3.1 times higher risk of TB from SAM than their complements [AHR: 3.11 (1.91, 4.72)]. Furthermore, children with baseline pneumonia were twice as likely to acquire TB than children without pneumonia [AHR: 2.10 (1.76,12)]. In this study, the risk of TB among children with failed appetite taste at the diagnosis of SAM was almost 2.4 times higher [AHR: 2.41 (1.35, 3.82)]. The risk of TB also increased threefold for children who had HIV/AIDS compared with no cases [AHR: 3.71 (95% CI: 2.10, 8.71)]. (Table 3). The Kaplan-Meier failure estimates of the independent predictors were also drawn with Kaplan-Meier curves (Figure 4).

**Table 3:** Bivariable and multivariable Cox regression model showing the predictors of time to TB occurrence in under-five children with SAM admitted from January 20, 2014, to June 20, 2019, in north shoa, Ethiopia, Ethiopia.

Baseline Variables	Category	Outcome		CHR	AHR
		TB case	Censored		
Residency	Urban	9	114		
	Rular	15	207	0.97 (0.12,2.43)	1.21 (0.34.3.12)
Sex	Male	13	198		
	Female	11	123	0.82 (0.43,1.21)	0.45 (0.21,0.91)
Anemia	Yes	12	16	1.09 (1.01,3.12)	0.91 (0.67.2.12)
	No	12	8		
Vitamine A	Yes	10	150		
	NO	14	171	1.30 (1.10,4.31)	1.10 (0.85,1.89)
Immunization status	Yes	6	221		
	No	18	100	1.1 (0.94,2.31)	1.1 (0.81.2.41)
Ever breastfeed	Yes	6	154		
	No	18	167	1.2 (1.01,3.45)	0.94 (0.56.2.31)
NG tube	Yes	15	76	1.43 (0.93.2.54)	0.12 (0.01,1.23)
	No	9	245		
Baseline supplementation of zin	Yes	3	45		
	No	21	276	2.43 (2.03,4.02)	3.1 (1.91, 4.70) *
Appetite test	Pass	3	212		
	Failed	21	109	4.01 (3.07,5.70)	1.4 (1.05,3.82)*
ReSoMal solution	Yes	16	74	1.3 (1.10,3.09)	1.10 (0.83.2.11)
	No	8	247		
History of contact with TB patient	Yes	18	111	2.45 (1.23,4.53)	1.68 (1.11,3.12)*
	No	6	210		
HIV/AIDS	Yes	15	8	2.68 (2.01,10.87)	3.71 (2.10, 8.71)*
	No	9	313		
Baseline pneumonia	Yes	20	88	4.51 (3.23, 6.78)	2.10 (1.76,12)*
	No	4	233		

* *p* value ≤0.05.

HIV/AIDS, Human Immunodeficiency Virus/Acquired Immunodeficiency Syndrome.

## Discussion

The current finding aimed to assess the incidence of TB and its predictors among children under five years of age with SAM in North Shoa, Amhara, Ethiopia. According to the current study, the incidence density rate of TB among under-five children with SAM was 4.6 per 100 child months (95% CI: 7.29, 8.9). Similarly, the proportion of TB in under-five children with SAM was 7% (95% CI: 5.3–9.1). The finding is congruent with a study conducted in Ethiopia: pawi general hospital (6.8%) (23), in Gondar (9.7%) (24), (9.6%) (25), in India (8.8%) (26) and Bangladesh (7%) (27). However, our findings are less than those of a study conducted in Felege-Hiwot Referral Hospital (16.2%) (28) and Yekatit 12 Hospital (20.8% (20). A finding from Freetown (20%) (29) and India (22%) (22) was also higher than in this study. This difference might be attributed to varying screening strategies, access to TB diagnostics, and varying clinical access to diagnostic tests for childhood TB diagnosis and treatment among hospitalized children. However, our finding is higher than a study conducted in Zambia (1.58)% (21) and in Kenya (4.2%) (30). This could be due to a gap in practicing policy in implementing guidelines, capacity building at the country level, and decentralizing and integrating TB services into broader child health programs. In this retrospective follow-up study, the overall free survival of TB was 99.4%, 92.3% and 82.9% at 1, 13 and 24 months, respectively.

This study found that there were multiple predictors of TB in under-five children with SAM in North Shoa, Amhara region, Ethiopia, including having a history of contact with known TB cases, no supplementation of zinc at the baseline, failed appetite taste at the diagnosis of SAM, HIV/AIDS and baseline pneumonia, which were found to be inversely related to the time to TB occurrence in under-five children with SAM. Thus, children with a contact history with known TB cases had a nearly two times higher risk of contracting TB than their counterparts. This is supported by a study performed in China (31). Likewise, children with baseline pneumonia as a comorbidity along with SAM were at higher risk of acquiring TB earlier than those who did not have pneumonia. This is supported by a study in Uganda and a systematic review done in Africa (32, 33).

Furthermore, children who did not take baseline zinc supplementation as part of the SAM treatment had a nearly three-fold higher risk of TB than their counterparts. This could be explained by the fact that zinc is vital to boost the immune system and epithelialization of the lung tissues. In this study, the probability of getting TB among children with failed appetite taste was almost two and a half times higher, as in a study in Niger (34). Furthermore, the risk of TB also increased threefold for children who had HIV/AIDS compared with no cases. A similar finding was reported in Tanzania (35). This could be because HIV/AIDS suppress the immune system, thereby increasing the probability that latent TB will develop into an active disease (22–24). The predictor of diarrhea causes statistical problems in the final model, which might cause separation. Separation occurs when a predictor variable perfectly predicts the outcome variable, resulting in an infinite estimate of the predictor's effect. This means that there would be a subset of observations in which the predictor variable (diarrhea) always has the same value for each observation, and all of these observations have the same outcome value (diarrhea). In this case, the regression model would be unable to estimate the effect of other predictors in the presence of diarrhea because the model would be over-reliant on diarrhea as the sole predictor. Therefore, it is essential to carefully consider the quality and quantity of predictor variables in a regression model. By addressing issues such as inadequate sample size, the inadequate definition of predictor variables, and the need to collapse variables, we tried to improve the reliability and validity of the findings and draw more accurate conclusions about the relationships between predictors and outcome variables. In

conclusion, the outcomes of this study reveal that the incidence of TB among under-five children with SAM was found to be considerably higher in the study area. In addition, having a history of contact with known TB cases, not having a baseline supplementation of zinc, failed appetite taste at the diagnosis of SAM, and baseline pneumonia were found to be a risk factor for TB among under-five children with SAM. Thus, based on these findings, health care workers should focus on providing nutritional support to under-five children with SAM, prioritizing regular TB screenings and assessments, supporting children with failed appetite taste and pneumonia, and encouraging zinc supplementation to prevent the development of TB.

### Strength and limitation of the study

We used a standard data extraction tool adopted from national TB and malnutrition management guidelines of the Federal Ministry of Health of Ethiopia. Long-term follow-up period, in which we can draw observational conclusions. Additionally, this study will add a new input to the area since findings are scarce. Since the data were collected from secondary sources, some significant predictors might need to be included. Some of the predictors are also difficult to define due to the nature of secondary data and unmeasured issues during data collection period. Careful interpretation is highly recommended. This study could not use controls for comparison, which is a limitation.

## Data Availability

The original contributions presented in the study are included in the article/supplementary material, further inquiries can be directed to the corresponding authors.
